# Capecitabine plus Irinotecan (XELIRI regimen) compared to 5-FU/LV plus Irinotecan (FOLFIRI regimen) as neoadjuvant treatment for patients with unresectable liver-only metastases of metastatic colorectal cancer: a randomised prospective phase II trial

**DOI:** 10.1186/1471-2407-9-120

**Published:** 2009-04-22

**Authors:** Erik Skof, Martina Rebersek, Zvezdana Hlebanja, Janja Ocvirk

**Affiliations:** 1 Institute of Oncology, Division of Medical Oncology, Zaloska 2, 1000 Ljubljana, Slovenia

## Abstract

**Background:**

Phase II studies have shown that the combination of capecitabine and irinotecan (the XELIRI regimen) is active in metastatic colorectal cancer (MCRC). There are, however, no data about the use of the XELIRI regimen in the neoadjuvant treatment.

**Methods:**

Patients with unresectable liver-only metastases of MCRC with ≤ 75 years of age were randomised to either the XELIRI (irinotecan 250 mg/m^2 ^given on day one and capecitabine 1000 mg/m^2 ^twice daily from day 2–15, every 21 days) or the FOLFIRI arm (irinotecan 180 mg/m^2^, 5-FU 400 mg/m^2^, LV 200 mg/m^2^, 5-FU 2400 mg/m^2 ^(46-h infusion) – all given on day one, every 14 days). Primary end points were objective response rate (ORR) and rate of radical (R0) surgical resection. Secondary end points were progression-free survival (PFS), overall survival (OS) and safety.

**Results:**

Altogether 87 patients were enrolled (41 pts in the XELIRI and 46 pts in the FOLFIRI arm). The median age was 63 years (63 years in the XELIRI and 62 years in the FOLFIRI arm) (p = 0.33). ORR was 49% in the XELIRI and 48% in the FOLFIRI arm (p = 0.76). The rate of radical R0 resection was 24% in both arms of patients. At the end of treatment, 37% of patients in the XELIRI and 26% of patients in the FOLFIRI arm were without evidence of the disease (CR+R0 resection) (p = 0.56). There were no statistical differences in grade 3 or 4 adverse events between both arms: diarrhoea 7% vs. 6%, neutropenia 5% vs. 13%, ischemic stroke 0 vs. 2%, acute coronary syndrome 2% vs. 4%, respectively. At the median follow up of 17 (range 1–39) months, the median PFS was 10.3 months in the XELIRI and 9.3 months in the FOLFIRI arm (p = 0.78), the median OS was 30.7 months in the XELIRI arm and 16.6 months in the FOLFIRI arm (p = 0.16).

**Conclusion:**

The XELIRI regimen showed similar ORR as the FOLFIRI regimen in the neoadjuvant treatment of patients with MCRC. In addition, the XELIRI regimen showed similar PFS and OS with acceptable toxicity compared to the FOLFIRI regimen.

**Trial Registration:**

Current Controlled Trials ISRCTN19912492

## Background

The majority of patients with metastatic colorectal cancer (MCRC) have liver metastases. Radical (R0) resection of liver metastases offers the greatest likelihood of cure in the patients with liver-only metastases of MCRC. The five-year survival rates after radical resection are 24–58% [1-4], whereas the five-year survival rates for unresectable disease with most active systemic chemotherapy regimens are <5% [5]. Since the majority of patients with MCRC have unresectable liver metastases, neoadjuvant (or preoperative) chemotherapy is an appropriate treatment choice with intent to reduce the number and/or size of the liver metastases to make the radical (R0) resection of liver metastases possible. This can be achieved in 12–33% of patients [6-8]. In the neoadjuvant chemotherapy, combinations of fluoropyrimidines (5-FU/leucovorin (LV)) with irinotecan and/or oxaliplatin are usually used. Among the combinations of fluoropyrimidines with irinotecan, the FOLFIRI regimen is preferred, whereas the FOLFOX regimen is preferred among the combinations of fluoropyrimidines with oxaliplatin. Randomised studies have shown that the combination of capecitabine with oxaliplatin (XELOX regimen) in the first-line treatment of patients with MCRC shows similar efficacy and tolerability compared to the combinations of 5-FU/LV with oxaliplatin [9,10]. There are, however, only few published data about the combination of capecitabine with irinotecan in the treatment of patients with MCRC. Phase I/II studies have demonstrated that the combination of capecitabine (1000 mg/m2 twice daily for 14 days) with irinotecan (250 mg/m2 i.v. on day one, every 21 days) (the XELIRI regimen) is active [11-14]. The XELIRI regimen showed response rates of 35–54% and time to progression 8–9 months. Most common treatment-related grade 3 or grade 4 adverse events reported from a phase II study were neutropenia (25%), diarrhoea (20%), vomiting (16%), dehydration (10%), nausea (6%), abdominal pain (6%), and hand-foot syndrome (6%) [11].

To our knowledge, there has been no study published with the XELIRI regimen in the neoadjuvant setting of patients with unresectable liver-only metastases of MCRC. The aim of our study was to compare the efficacy, safety and survival of the XELIRI regimen to the standard FOLFIRI regimen in the neoadjuvant setting of patients with unresectable liver-only metastases of MCRC.

Our hypothesis was that there are no statistically significant differences in efficacy, survival and safety of the XELIRI regimen compared to the standard FOLFIRI regimen. However, the XELIRI regimen seems to be more convenient when compared to the FOLFIRI regimen since capecitabine is an oral drug; therefore, there is no need for central venous catheters implantation (risk for bleeding, infection, thrombosis) and no need for hospitalisation. The treatment is performed in out-patient clinic. The cycles are applied every 21 days compared to every 14 days in the FOLFIRI regimen.

In the year 2004, at the time when our study was initiated, bevacizumab was not yet registered for the treatment of patients with MCRC in Slovenia. However, in 2006, while our study was ongoing, bevacizumab became a standard in the first-line treatment of patients with MCRC. This was the main reason why the study was prematurely closed for the accrual at the end of the year 2006 with only 43% of initially planned accrual.

## Methods

The study was performed at the Institute of Oncology Ljubljana, Slovenia, after it had been approved by National Medical Ethics Committee. This was a prospective randomised phase II study.

***Eligibility criteria were***: age 18–75 years, performance status of 0–1 according to WHO, unresectable liver metastases of colorectal adenocarcinoma – determined by liver surgeon either because of the size, number, or unfavourable location of metastases that did not allow a complete resection of disease leaving at least 25% of normal liver parenchyma, no prior chemotherapy for metastatic disease, >6 months since adjuvant treatment, at least one measurable lesion of ≥ 1 cm visible on spiral computed tomography (CT), bilirubin < 2× times the upper limit of normal (ULN), aspartate aminotransferase (AST) < 5× ULN, alanine aminotransferase (ALT) < 5× ULN, adequate haematological and renal function, and signed informed consent.

***Exclusion criteria were***: extra-hepatic disease (either metastases outside liver or loco-regional recurrence), other malignancy within the past 5 years (except limited basal cell or squamous cell carcinoma of the skin or in situ cervical carcinoma), inadequately controlled hypertension (blood pressure >150/100 mmHg on antihypertensive medications), unstable angina pectoris, history of myocardial infarction or stroke within 6 months, clinically significant peripheral vascular disease, inflammatory bowel disease. Patients that fulfilled eligibility criteria were randomly assigned to either the XELIRI or FOLFIRI arm with no prior stratification.

### Patient evaluation

Pre-treatment evaluation included a detailed medical history, physical examination, a complete blood count with differential and platelet count, blood chemistry, serum levels of carcinoembryonic antigen (CEA) and baseline tumour measurements by CT. The pre-treatment evaluation had to be performed within 14 days before the treatment was initiated. The patients were assessed for toxicity before each application of cytotoxic drugs. Repeat imaging was required every three months during treatment. Chemotherapy was discontinued prior to the expiry of six months if liver metastases became resectable (patients were referred to liver surgeon) or if there was either progression of the disease or serious adverse event occurred during chemotherapy. After the discontinuation of study treatment, a follow-up examination, including clinical examination, blood samples (liver enzymes, CEA), CT or ultrasound of the abdomen, was performed every 3 months, until the progression of the disease or death.

### Chemotherapy

The XELIRI regimen consisted of irinotecan 250 mg/m^2 ^given on day one and capecitabine 1000 mg/m^2 ^twice daily from day 2–15, every 21 days. The FOLFIRI regimen consisted of irinotecan 180 mg/m^2^, 5-FU 400 mg/m^2^, LV 200 mg/m^2^, 5-FU 2400 mg/m^2 ^(46-h infusion) – all given on day one, every 14 days. The patients in both arms received premedication with dexamethason 20 mg i.v., granisetron 1 mg i.v. and diazepam 10 mg i.v. on day 1 of each chemotherapy cycle. To initiate a cycle of cytotoxic chemotherapy, an absolute neutrophil count of at least 1500/μl, platelets at least 100000/μl and resolution of other toxic effects to at least CTC grade 1 were required. A resolution of toxicity to at least CTC grade 1 was required within 3 weeks of the intended start of a cycle or patients were withdrawn from the study. All patients were advised to use emollients with urea to manage hand-foot syndrome of grade I and II. The maximum planned duration of the treatment was six months in both arms. Postoperative chemotherapy was not planned.

### Surgery

At evaluation, surgical resection of metastases was reconsidered by a team of experts, consisting of a liver surgeon, radiologist and medical oncologist. Liver surgery was attempted when technically feasible and potentially curative in the patients fit for operative procedure. During surgery, a complete exploration of the abdomen, including intraoperative ultrasound, was performed. Different surgical techniques were used for the resection of metastases.

***The primary end points ***were: overall objective response (ORR) and rate of radical surgical resection (R0 resection). The evaluation of response was based on RECIST [15]. The radical surgical resection (R0 resection) of liver metastases was defined as tumour-free margin of >10 mm at histology specimen and no signs of residual metastases during exploration of the abdomen. In case of complete response (CR), liver surgery was not performed; regular follow-up was performed in these patients.

***Secondary end points ***were: progression-free survival (PFS), overall survival (OS) and safety. PFS was defined as the time between the randomisation and the progression of the disease or death of any cause; the patients who were withdrawn from the study treatment for other reasons were censored at the discontinuation of study therapy. OS was defined as the time between the randomisation and death. Treatment-related death was defined as the death within 30 days after the last cycle of chemotherapy or liver surgery. All toxic effects were graded according to the National Cancer Institute (NCI) – Common Toxicity Criteria (CTC), version 3.0. The histology reports documented any signs of steatohepatitis.

### Statistical considerations

For the statistical power calculation, online DSS Research toolkit was used. For the FOLFIRI arm, a response rate of 40% and a R0 resection rate of 15% was expected. The statistical power for initially planned 200 patients, with Alpha Error level of 5% (a 95% Confidence Interval) to show 10% difference between arms, was calculated to be 41% for the objective response rate, and 55% for the R0 resection rate. With 87 patients enrolled, the statistical power for the objective response rate was 24%, and for the R0 resection rate 32%.

Statistical analysis was performed with the program SPSS – Version 1.3. For the comparison of the two arms, two-sided Pearson chi-square test and Student's t-test were used. Objective response rate was assessed on intention to treat (ITT) population. Safety analysis was performed on the group of patients who received at least one dose of protocol medication. Overall survival and progression-free survival were estimated according to Kaplan-Meier method [16]. Survival curves were compared with the log-rank test. Statistical difference between the arms was determined as p < 0.05.

## Results

In the period of 1 January 2004 – 31 December 2006, altogether eighty-seven patients were enrolled. Forty-one patients were randomly assigned to the XELIRI and forty-six patients to the FOLFIRI arm. There were no statistically significant differences in baseline characteristics between the two arms (Table 1). Median duration of neoadjuvant treatment was 5.0 (range 1.1 – 9.6) months in the XELIRI arm and 5.1 (range 0.1 – 9.7) in the FOLFIRI arm (p = 0.45). The median follow up was 17 months (range 1–39). The efficacy of both treatment arms (ORR, R0 resection rate) is shown in Table 2. At the time of evaluation by CT scans after the conclusion of neoadjuvant chemotherapy, the disease was expected to be resectable in 29% of patients in the XELIRI arm and in 44% of patients in the FOLFIRI arm (p = 0.16). All of these patients underwent surgery. The R0 resection has been performed in 10 (24%) of all patients in the XELIRI arm and in 11 (24%) of all patients in the FOLFIRI arm (p = 0.83).

**Table 1:** Baseline clinical characteristics of patients in the XELIRI and FOLFIRI arms, data are shown as n (%) or as n (range)

	XELIRI	FOLFIRI	
	n = 41	n = 46	p
**Median age-years**	63 (47–75)	62 (34–75)	0.33
**Gender**			
Male	26 (63%)	27 (59%)	0.38
Female	15 (37%)	19 (41%)	0.40
**WHO performance status**			
Performance status 0	31 (75%)	36 (78%)	0.82
Performance status 1	10 (25%)	10 (22%)	0.85
**Primary tumour**			
Colon	30 (73%)	40 (87%)	0.23
Rectum	11 (27%)	6 (13%)	0.22
**Initial stage of disease at diagnosis**			
Stage 1	0	1 (2%)	-
Stage 2	7 (17%)	3 (7%)	0.21
Stage 3	6 (15%)	14 (30%)	0,07
Stage 4	28 (68%)	28 (61%)	1.0
Previous adjuvant treatment*			
Yes	6 (15%)	10 (22%)	0.32
No	35 (85%)	36 (78%)	0.91
**Median time from diagnosis to randomisation (months)**	1.87 (0.7–65.5)	2.1 (0.26–55.7)	0.77
**Number of liver metastases**			
1 – 4	15 (37%)	11 (24%)	0.43
5 – 10	8 (19%)	10 (22%)	0.81
> 10	18 (44%)	25 (54%)	0.28
**Liver involvement (%)**			
< 25	14 (34%)	10 (22%)	0.41
25 – 50	13 (32%)	14 (30%)	0.85
> 50	14 (34%)	21 (45%)	0.24
**Median size of liver metastases (cm)**	4.0 (1.5 – 12.4)	5.0 (0.5–15.0)	0.10
**Bilateral liver metastases, n (%)**	35 (85%)	37 (80%)	0.81
**Baseline CEA**			
Normal	8 (19%)	10 (22%)	0.64
> 3.5 μg/l	33 (81%)	36 (78%)	0.72
**Baseline LDH**			
Normal	25 (61%)	18 (39%)	0.28
> 4.12 μkat/l	15 (37%)	26 (56%)	0.09
**Reasons for initial unresectability**			
Location of metastases	10 (24%)	8 (17%)	0.52
Number of metastases	25 (61%)	30 (65%)	0.71
Size of metastases	6 (15%)	8 (17%)	0.84

* Adjuvant treatment with chemotherapy ± radiotherapy

**Table 2:** Efficacy of treatment in the XELIRI and FOLFIRI arms (response rate, R0 resection* rate), data are shown as n (%)

	XELIRI	FOLFIRI	
	n = 41	n = 46	p
**Complete response**	5 (12%)	1 (2%)	0.10
**Partial response**	15 (37%)	21 (46%)	0.32
**Objective response**	20 (49%)	22 (48%)	0.76
**Stagnation**	12 (29%)	10 (22%)	0.67
**Progressive disease**	7 (17%)	11 (24%)	0.35
R0 resection*	10 (24%)	11 (24%)	0.83
**Complete response + R0 resection**	15 (37%)	12 (26%)	0.56

R0 resection* – > 10 mm tumour-free resection margin on histology

**Figure 1:**
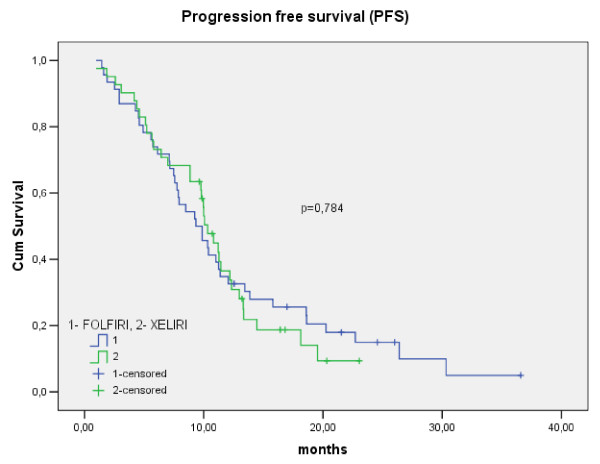
**The PFS of patients in the XELIRI and FOLFIRI arms (months)**.

**Figure 2:**
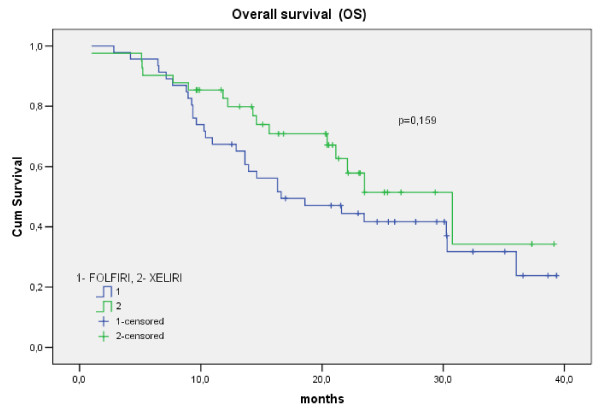
**The OS of patients in the XELIRI and FOLFIRI arms (months)**.

The PFS of patients in the XELIRI and FOLFIRI arms is shown in Figure 1. The median PFS for the patients in the XELIRI arm was 10.3 months (95% CI: 9.1–11.5) and 9.3 months (95% CI: 6.7–12.0) in the FOLFIRI arm (p = 0.78). The OS of patients in the XELIRI and FOLFIRI arms is shown in Figure 2. The median OS for the XELIRI arm was 30.7 months (95% CI: 19.5–41.9) and for the FOLFIRI arm 16.6 months (95% CI: 7.9–25.3) (p = 0.16).

At the time of the analysis, of 21 patients who underwent R0 resection of liver metastases, 19 (90%) were alive: in the XELIRI arm, 9 out of 10 (90%) patients, and in the FOLFIRI arm, 10 out of 11 (91%) (p = 0.82). At the time of the analysis, CR after chemotherapy was evident in 4 out of 6 (67%) patients: in the XELIRI arm, 3 out of 5 (60%) patients, and in the FOLFIRI arm, 1/1 patient (p = 0.32).

Adverse events of any grade in the treatment the XELIRI and FOLFIRI arms are shown in Table 3. The majority of adverse events were of grade I or II. The adverse events of grade 3 or 4 in the XELIRI arm were diarrhoea (7%), neutropenia (5%), and acute coronary syndrome (2%). In the FOLFIRI arm, the adverse events of grade 3 or 4 were neutropenia (13%), diarrhoea (6%), acute coronary syndrome (4%), and ischemic stroke (2%). No hand-foot syndrome of grade 3 occurred in any of the two arms. Dose reduction due to adverse events was required in 12% of patients in the XELIRI arm and in 22% of patients in the FOLFIRI arm (p = 0.20). The therapy was completed as planned in 90% of patients in the XELIRI arm and in 87% of patients in the FOLFIRI arm (p = 0.85). The diagnosis of steatohepatitis from histology reports at liver surgery was present in 6/12 (50%) of patients in the XELIRI arm and in 8/20 (40%) of patients in the FOLFIRI arm (p = 0.59). Steatohepatitis was present in median of 65% (range 20% – 90%) of liver tissue in the XELIRI arm and in median of 55% (range 10% – 70%) in the FOLFIRI arm (p = 0.59). The intensity of steatohepatitis was either mild (3 patients) or modest (3 patients) in the XELIRI arm, and mild (5 patients), modest (1 patient) and severe (2 patients) in the FOLFIRI arm (p = 0.30). None of patients with steatohepatitis had any additional perioperative co-morbidity after liver surgery. There were no treatment-related deaths in any of the two arms of patients.

**Table 3:** Adverse events regarding CTCAE-3 in the XELIRI and FOLFIRI arms, data are shown as n (%)

	XELIRI	FOLFIRI	
	N = 41	n = 46	p
**Hand-foot syndrome**			
Grade 1	4 (10%)	0	0.18
Grade 2	2 (5%)	0	0.10
Grade 3	0	0	-
**Diarrhoea**			
Grade 1	1 (2%)	4 (8%)	0.18
Grade 2	4 (10%)	7 (15%)	0.37
Grade 3	1 (2%)	2 (4%)	0.56
Grade 4	2 (5%)	1 (2%)	0.56
**Nausea**			
Grade 1	3 (7%)	8 (17%)	0.13
Grade 2	6 (15%)	1 (2%)	0.06
Grade 3	0	0	-
Grade 4	0	0	-
**Fatigue**			
Grade 1	3 (7%)	4 (9%)	0.70
Grade 2	1 (2%)	2 (4%)	0.56
Grade 3	0	0	-
Grade 4	0	0	-
**Neutropenia**			
Grade 1	3 (7%)	2 (4%)	0.65
Grade 2	2 (5%)	12 (26%)	0.008
Grade 3	1 (2%)	5 (11%)	0.10
Grade 4	1 (2%)	1 (2%)	1.0
**Thrombopenia of any grade**	0	0	-
**Ischemic stroke**	0	1 (2%)	-
**Acute coronary syndrome**	1 (2%)	2 (4%)	0.56
Death*	0	0	-

* Patients who died within 30 days after the last cycle of chemotherapy or liver surgery

At relapse, all patients received at least one line of chemotherapy: oxaliplatin-based second-line therapy received 85% of patients in the XELIRI arm and 62% of patients in the FOLFIRI arm (p = 0.59), cetuximab-based second-line therapy received 5% of patients in the XELIRI arm and 12% of patients in the FOLFIRI arm (p = 0.32).

## Discussion

The present study reports the comparison of efficacy, safety and survival between the XELIRI and FOLFIRI regimen in the neoadjuvant treatment of patients with MCRC.

The current single-institution randomised phase II trial demonstrated that the XELIRI regimen is effective in the neoadjuvant treatment of patients with MCRC and that it has acceptable toxicity, when compared to the FOLFIRI regimen. Both primary end-points were practically identical between the two arms. The relatively high rate of objective response (49% and 48%) in both arms is in the range of expected, since all patients had liver-only metastases. The rate of R0 resection (24%) of liver metastases in our study is one of the highest reported. We believe that both chemotherapy regimens are effective since, at the end of treatment, 37% of patients were without any evidence of the disease (either complete remission achieved with chemotherapy or R0 resection of liver metastases) in the XELIRI regimen and 26% of patients in the FOLFIRI regimen.

The PFS of 9–10 months in the patients in our study was similar to the PFS of patients with liver only metastases treated with the FOLFIRI regimen in a recently reported large randomised the CRYSTAL trial [17]. In the CRYSTAL trial, however, the secondary resection rate of liver metastases in 134 patients with liver-only metastases was only 4.5%. Since the majority of patients in the CRYSTAL trial had extra-hepatic disease, it is possible that the low rate of secondary resection of liver-only metastases was due to the lack of active searching for resectable liver metastases after neoadjuvant chemotherapy by the multidisciplinary team consisting of a liver surgeon, radiologist and medical oncologist. We believe that this multidisciplinary approach is essential for an optimal treatment outcome of patients with liver-only metastases of MCRC.

Despite the high R0 resection rate in both our study arms and high survival of patients following R0 resection in our study, the median OS of all patients in the FOLFIRI arm is one of the lowest ever reported for this regimen (16.6 months). It is possible that poor median OS in our FOLFIRI arm is due to the fact that only 62% of patients in this arm received an oxaliplatin-based second line regimen at progression of the disease. For the patients with MCRC who receive all three chemotherapeutic drugs (irinotecan, oxaliplatin, and fluoropyrimidine), the expected median OS is 20–22 months [18,19].

It is noteworthy that the XELIRI regimen showed acceptable toxicity. To our surprise, no grade 3 hand-foot syndrome, a well-known adverse event of capecitabine, occurred in the XELIRI arm. This is possibly due to the lower daily dose of capecitabine in the XELIRI regimen than in the capecitabine monotherapy. During the treatment with irinotecan and capecitabine, diarrhoea is a frequent adverse event when these drugs are used as single agents. In our study, the rate of grade 3 or 4 diarrhoea in the XELIRI arm was also low. This is in concordance with the European CAIRO trial [20], which showed no additional grade 3 or 4 diarrhoea in the patients in the XELIRI arm as compared to the sequential therapy with capecitabine and irinotecan monotherapy. In contrast, in the recently published American trial BICC-C [21], the incidence of grade 3 or 4 diarrhoea was present in 48% of patients in the XELIRI arm. The reason for these differences in diarrhoea incidences among these trials is not known.

The neoadjuvant chemotherapy is known to cause liver toxicity. For the irinotecan-based regimens, the usual pathological abnormality of liver cells is steatohepatitis. Because of liver toxicity, the resection of liver metastases should be performed as soon as the metastases become resectable with neoadjuvant chemotherapy. There were signs of steatohepatitis in both arms of patients, but no additional co-morbidities were observed after liver resections in the patients of either arm.

In the recent years, three targeted drugs became available for the treatment of patients with MCRC (bevacizumab, cetuximab, panitumumab). Since best clinical benefit of these drugs is achieved in combination with chemotherapy, they are usually used with different chemotherapy regimens. The combination of targeted therapy with different chemotherapy regimens showed favourable objective response rates when compared to chemotherapy alone in phase II/III studies [17,22]. In the neoadjuvant setting, the regimens with higher objective response rates are preferred as it is believed that higher objective response rates lead to higher R0 resection rates of liver metastases. Therefore, the combination of targeted agents with chemotherapy is generally applied in the neoadjuvant setting. Because of this, our study was prematurely closed soon after bevacizumab had become available in our country. We believe that the XELIRI regimen should be considered to be part of phase II trials where the targeted drugs, like cetuximab or bevacizumab, are used in combination with chemotherapy. Outside clinical trials, in small subset of patients, where the targeted agents are contraindicated or not available to the patients, the XELIRI regimen could be used as an alternative to the FOLFIRI regimen in the neoadjuvant setting of patients with liver-only metastases of MCRC.

## Conclusion

Our study showed that the XELIRI regimen is effective with acceptable toxicity when compared to the FOLFIRI regimen in neoadjuvant treatment of patients with unresectable liver-only metastases of MCRC. XELIRI regimen merits further evaluation in phase II trials in combination with targeted drugs in patients with MCRC.

## Competing interests

The authors declare that they have no competing interests.

## Authors' contributions

ES made a substantial contribution to the conception, design, and coordination of the study, to the treatment of patients, interpretation of data; he performed statistical analysis, and was involved in drafting the manuscript. MR made a substantial contribution to the treatment of patients and helped to draft the manuscript. ZH made a substantial contribution to the treatment of patients and helped to draft the manuscript. JO made a substantial contribution to the conception, design and coordination of the study and to the treatment of patients, and helped to draft the manuscript. All authors read and approved the final manuscript.

## Pre-publication history

The pre-publication history for this paper can be accessed here:

http://www.biomedcentral.com/1471-2407/9/120/prepub
